# Interaction and uptake of exosomes by ovarian cancer cells

**DOI:** 10.1186/1471-2407-11-108

**Published:** 2011-03-27

**Authors:** Cristina Escrevente, Sascha Keller, Peter Altevogt, Júlia Costa

**Affiliations:** 1 Instituto de Tecnologia Química e Biológica, Apartado 127, 2781-901 Oeiras, Portugal; 2 Tumor Immunology Programme, D015-TP3, German Cancer Research Center, Heidelberg, Germany

## Abstract

**Background:**

Exosomes consist of membrane vesicles that are secreted by several cell types, including tumors and have been found in biological fluids. Exosomes interact with other cells and may serve as vehicles for the transfer of protein and RNA among cells.

**Methods:**

SKOV3 exosomes were labelled with carboxyfluoresceine diacetate succinimidyl-ester and collected by ultracentrifugation. Uptake of these vesicles, under different conditions, by the same cells from where they originated was monitored by immunofluorescence microscopy and flow cytometry analysis. Lectin analysis was performed to investigate the glycosylation properties of proteins from exosomes and cellular extracts.

**Results:**

In this work, the ovarian carcinoma SKOV3 cell line has been shown to internalize exosomes from the same cells via several endocytic pathways that were strongly inhibited at 4°C, indicating their energy dependence. Partial colocalization with the endosome marker EEA1 and inhibition by chlorpromazine suggested the involvement of clathrin-dependent endocytosis. Furthermore, uptake inhibition in the presence of 5-ethyl-N-isopropyl amiloride, cytochalasin D and methyl-beta-cyclodextrin suggested the involvement of additional endocytic pathways. The uptake required proteins from the exosomes and from the cells since it was inhibited after proteinase K treatments. The exosomes were found to be enriched in specific mannose- and sialic acid-containing glycoproteins. Sialic acid removal caused a small but non-significant increase in uptake. Furthermore, the monosaccharides D-galactose, α-L-fucose, α-D-mannose, D-N-acetylglucosamine and the disaccharide β-lactose reduced exosomes uptake to a comparable extent as the control D-glucose.

**Conclusions:**

In conclusion, exosomes are internalized by ovarian tumor cells via various endocytic pathways and proteins from exosomes and cells are required for uptake. On the other hand, exosomes are enriched in specific glycoproteins that may constitute exosome markers. This work contributes to the knowledge about the properties and dynamics of exosomes in cancer.

## Background

Exosomes are small membrane vesicles between 40-100 nm in diameter that are secreted by various cell types, including tumor cells, neurons, B- and T- lymphocytes, intestinal epithelial cells [1-3] and in physiological fluids [3,4].

Exosome biogenesis involves the inward budding of endosomes into multivesicular bodies to form intraluminal vesicles that are then released to the extracellular space. During this process transmembrane proteins are incorporated into the invaginating membrane maintaining the same topological orientation as the plasma membrane, while cytosolic components are engulfed.

The molecular basis of protein sorting during exosomes formation appears to involve a ubiquitin-dependent mechanism and the endosomal sorting complexes required for transport (ESCRT) [5,6]. However, some proteins present in the exosomes are not ubiquitinated suggesting that other mechanisms such as oligomerization or partitioning of protein into lipid raft domains may be involved [6-9].

Exosomes are released by multivesicular bodies fusion with the plasma membrane and the mechanism appears to involve Rab27A and Rab27B [10]. Recent studies have suggested their participation in different physiological and/or pathological processes, such as, tumor progression, stimulation of the immune system, coagulation and inflammation and intercellular transfer of infectious agents, such as proteins and RNA [4,6,8].

Many of the functions described for exosomes depend on their ability to specifically interact with a target cell and several types of interaction have already been proposed based on indirect evidence and *in vitro *studies. Exosomes can associate with the plasma membrane through ligand-receptor interactions [11] or lipids, such as phosphatidylserine [12]. The process of internalization can occur through direct fusion of the exosomes with the plasma membrane, leading to the release of the exosomal content into the cell cytoplasm. Alternatively, exosomes can enter the cells by receptor-mediated endocytosis and later fuse with the limiting membrane of the endosome releasing the exosomal content to be recycled to the cell surface or to be degraded in the lysosome [11,13]. Exosome uptake was shown to occur via clathrin-mediated endocytosis in dendritic cells [14], as well as phagocytosis in monocytes and macrophages [15].

Exosomes have a unique protein and lipid composition that varies depending on the cells from which they originate, nevertheless, as a consequence of their endosomal origin nearly all exosomes contain proteins involved in membrane transport and fusion (RabGTPases, annexins, flotilin), in multivesicular bodies biogenesis (TSG101, Alix), heat shock proteins (Hsc70, Hsc90), integrins and tetraspanins (CD9, CD63, CD81, CD82) [16,17]. In addition, they are enriched in raft-lipids such as cholesterol, sphingolipids and ceramide. In exosomes, an enrichment of certain glycosylated motifs has also been observed [18].

Glycosylation is a post-translational modification that plays an important role in several properties of proteins including stability, folding, intracellular trafficking and recognition. Lectins and their interactions with carbohydrates have been found to play a role in exosome uptake by dendritic cells [19] and macrophages [20].

In the present work, the SKOV3 ovarian carcinoma cell line has been shown to internalize exosomes derived from the same cells via various endocytic pathways. Proteins from exosomes and from cells were required for the uptake. On the other hand, exosomes were highly enriched in specific glycosylated sialic acid- and mannose-containing glycoproteins, and sialic acid removal caused a small though non-significant increase in uptake.

## Methods

### Cell culture

Human ovarian cancer SKOV3, embryonic kidney HEK293 and neuroglioma H4 cell lines were grown in Dulbecco's Modified Eagle Medium (DMEM) (Sigma) at 37°C, 5% CO_2 _supplemented with 10% fetal calf serum (Gibco), 1% penicillin/streptomycin solution (Gibco).

### Isolation of secreted membrane vesicles

Confluent SKOV3, HEK293 and H4 cells were cultivated for 24 h in serum-free medium. The supernatant was collected and centrifuged, at 500, 10,000 and 100,000 × *g *10, 20 and 120 min, respectively, at 4°C. The pellet of the last centrifugation consisted of secreted membrane vesicles. Sucrose-density-fractionation was performed as described previously [21].

### Glycoprotein detection using lectins and immunoblot

Cellular extracts were obtained by solubilization of centrifuged cells in Triton X-100 buffer (50 mM Tris-HCl pH 7.5, 5 mM EDTA, 1% Triton X-100, 0.02% protease inhibitors cocktail, Complete, Roche), for 30 min. Total protein concentration was determined by the bicinchoninic acid (BCA) method.

Glycoproteins from total cellular extracts and secreted membrane vesicles were stained after transfer to polyvinylidene difluoride (PVDF) membrane with lectins. Concanavalin A (Con A) (Sigma), biotinylated *Sambucus nigra *(SNA) and *Maackia amurensis *lectin (MAL) (Galab Technologies) were used. Glycoproteins were fixed on the PVDF membrane with 25% (v/v) 2-propanol and 10% (v/v) acetic acid for 5 min. The membranes were blocked for 1 h with TBS, 0.1% Tween-20 (TTBS) for Con A or with TTBS containing 2% BSA for SNA and MAL. For Con A detection the membrane was incubated overnight with 25 μg/ml Con A in TTBS containing 1 mM CaCl_2 _and 1 mM MgCl_2 _(TTBSS) followed by 1 h incubation with 0.5 μg/ml horseradish peroxidase type I (Sigma) in TTBSS. For SNA and MAL detection, membranes were incubated overnight in TTBS with 0.5 or 5 μg/ml SNA or MAL lectin, respectively. Membranes were further incubated for 1 h with 0.02 μg/ml streptavidin-peroxidase (Sigma). Detection was performed with the Immobilon Western chemiluminescent HRP substrate (ECL) (Millipore).

Immunoblot was performed as previously described [22]. The following antibodies were used: mouse anti-CD9 (1:5000) and mouse anti-L1 (L1-11A) (1:3).

### Glycosidase treatment

Hydrolysis of α2,3- and α2,6-linked NeuAc from total protein cellular extracts and exosomes was carried out overnight at 37°C by the addition of 15 mU neuraminidase from *Vibrio cholerae *or from *Arthrobacter urefaciens *(Roche) in 50 mM sodium acetate pH 5.5 containing 4 mM CaCl_2_, and 50 mM sodium acetate pH 5.0, respectively. For specific hydrolysis of α2,3-linked NeuAc, 9 U neuraminidase from *Streptococcus pneumoniae *(Prozyme, Glyko) in 50 mM sodium phosphate pH 6.0 were used, for 1 hour at 37°C.

### Uptake of SKOV3 exosomes by SKOV3 cells

SKOV3 exosomes were labeled with carboxyfluoresceine diacetate succinimidyl-ester (CFSE) (Invitrogen) as previously described [12]. Briefly, exosomes (20 μg) collected after a 100,000 × g ultracentrifugation were incubated with 7.5 μM CFSE for 30 min at 37°C in a final volume of 200 μl PBS containing 0.5% BSA. Labeled exosomes (Exos-CFSE) were 65-fold diluted with DMEM supplemented with 10% vesicles-free fetal calf serum and pelleted by ultracentrifugation for 16 h at 10,0000 × g, 12°C. Exos-CFSE were resuspended in DMEM and incubated with SKOV3 cells at 37 or 4°C.

When indicated Exos-CFSE or cells were treated for 30 min with 100 μg/ml proteinase K, or for 2 h with 15 mU neuraminidase from *V. cholerae *or from *A. urefaciens *(Roche), before uptake. SKOV3 cells were also incubated, 30 min prior to and during uptake, with the inhibitors 10 μg/ml chlorpromazine, 5 μg/ml cytochalasin D, 50 μM 5-ethyl-N-isopropyl amiloride (EIPA) or 2% methyl-beta-cyclodextrin, or with 150 mM of the monosaccharides D-glucose, D-galactose, α-L-fucose, α-D-mannose, D-*N*-acetylglucosamine, and the disaccharide β-lactose (Sigma).

Uptake assays were always performed in the presence of the compounds and analyzed after 2 or 4 h by immunofluorescence microscopy or flow cytometry.

### Immunofluorescence microscopy

Immunofluorescence microscopy was done as previously described [22]. Primary antibodies were: mouse IgG anti-alpha-tubulin DM1A (1:2000) (Sigma), mouse IgG anti-EEA1 (1:100) (BD Biosciences), mouse IgG anti-LAMP1 H4A3 (1:100) (BD Biosciences) and mouse IgG anti-caveolin-1 (1:50) (Santa Cruz). Secondary antibody was donkey anti-mouse IgG AlexaFluor 594 (1:500) (Molecular Probes).

Images were acquired on a Leica DMRB microscope using a DFC340FX camera coupled to the microscope, and Leica application suite V3.3.0 software. For colocalization, images were acquired on a confocal SP5 microscope. Each picture was acquired with laser intensities and amplifier gains adjusted to avoid pixel saturation. Each fluorophore used was excited independently and sequential detection was performed. Each picture consisted of a z-series of 20 images of 1024-1024 pixel resolution with a pinhole of 1.0 airy unit. Colocalization analysis was performed using the open source Image J version 1.38 http://rsb.info.nih.gov/ij/.

### Flow cytometry

SKOV3 cells incubated with Exos-CFSE for 4 h at 37 or 4°C were washed with PBS, detached using trypsin and resuspended in PBS with 2% fetal calf serum (Gibco). Flow cytometry analysis was performed on a Cyflow ML cytometer (Partec) using Flowmax software 2.56 (Partec). Gate was set on living cells based on forward/side scatter properties and a minimum of 10^3 ^events within the gated live population were collected per sample. Exos-CFSE uptake by SKOV3 cells was measured by the shift in peak fluorescence intensity of CFSE, calculated by the geometric mean of the population. SKOV3 cells with no Exos-CFSE uptake (unlabelled) were used as controls for cell autofluorescence. Results were expressed as means ± S.D. and comparison between two means was performed by Student *t *test. A *P *value lower than 0.05 was considered significant.

## Results

### Uptake of SKOV3 exosomes by SKOV3 cells

**Figure 1:**
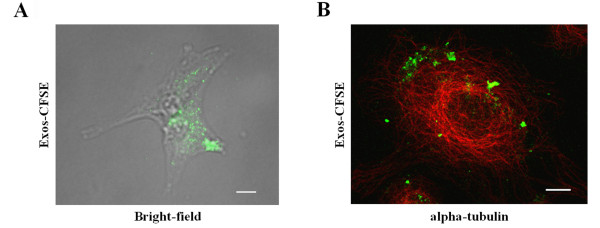
**Uptake of SKOV3 exosomes by SKOV3 cells**. (A) SKOV3 cells were incubated with Exos-CFSE (20 μg protein; green) for 4 h and were visualized in bright-field merged with fluorescence microscopy. Scale bar = 20 μm. (B) Detection of Exos-CFSE (green) and alpha-tubulin (red) by confocal immunofluorescence microscopy. Scale bar = 10 μm.

Since it has been shown that SKOV3 exosomes can be internalized by NK cells [12] here we have investigated if they are internalized by the cell line from where they originated. With that purpose exosomes produced in the absence of fetal bovine serum and collected at 100,000 × *g *were labeled with CFSE (Exos-CFSE) and incubated with SKOV3 cells. CFSE is a membrane permeable non-fluorescent compound that becomes fluorescent after cleavage of its acetate groups by intracellular esterases [23]. A punctuated green fluorescent pattern corresponding to Exos-CFSE interaction/internalization was observed after 30 min and it increased up to 4 hours of incubation (Figure 1A). To obtain a higher sensitivity exosomes were also biotinylated and detected with streptavidin Alexa-488, and the interaction was observed already 0.5 min after incubation (data not shown). Colocalization studies by immunofluorescence confocal microscopy of Exos-CFSE and alpha-tubulin, a microtubule marker, showed that Exos-CFSE were localized in the same z-stacks as microtubules, thus, confirming their internalization (Figure 1B). Supporting this conclusion was the observation that the exosomes were not removed by acid wash of cells pre-exposed to Exos-CFSE (data not shown).

**Figure 2:**
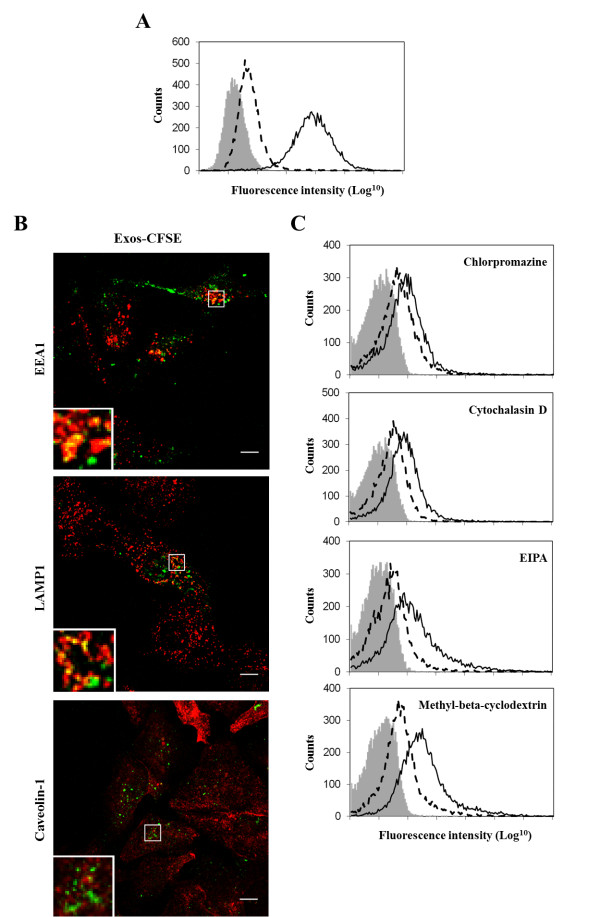
**Path of internalization of exosomes in SKOV3 cells**. (A) Exos-CFSE (20 μg protein; green) were incubated with SKOV3 cells at 37 or 4°C and uptake was monitored by flow cytometry analysis of cell fluorescence intensity. Solid and dashed lines represent Exos-CFSE uptake at 37 and 4°C, respectively. (B) Colocalization of Exos-CFSE (20 μg protein; green) with EEA1 (red), LAMP1 (red) and caveolin-1 (red). Secondary antibody was donkey anti-mouse IgG AlexaFluor 594. Colocalization is indicated in yellow. Images in the left bottom represent 4 × magnifications of selected areas. Scale bars = 10 μm. (C) Effect of chlorpromazine, cytochalasin D, EIPA and methyl-beta-cyclodextrin (30 min pre-incubation) on Exos-CFSE uptake (4, 4, 4 and 2 h, respectively) monitored by flow cytometry analysis (dashed lines). Controls consist of SKOV3 cells with no treatment for chlorpromazine and methyl-beta-cyclodextrin, or treated with DMSO for cytochalasin D and EIPA (solid lines). Unlabelled SKOV3 cells (grey) were used as control for cell autofluorescence. The results shown are representative of three independent experiments.

Several endocytic mechanisms have been described to mediate the entry of material into the cells [24]. Here, we observed that a decrease in temperature from 37 to 4°C caused a reduction of 80 ± 8% (n = 6) in the number of labeled cells as well as a decrease of 77 ± 9% (n = 6) in the uptake for the positively labeled cells, based on the shift in fluorescence intensity corresponding to the geometric mean of the population peaks (Figure 2A). These results indicated that exosomes uptake was mediated by endocytosis in an energy-dependent process. Exos-CFSE were also found to partially colocalize with the endosomal marker EEA1 (Figure 2B), thus showing the participation of clathrin-mediated endocytosis in exosome uptake. This conclusion was further supported by uptake inhibition (19 ± 18%, n = 4) (Figure 2C) with 10 μg/ml chlorpromazine, which blocks clathrin-mediated endocytosis [25]. Colocalization with the lysosomal marker LAMP1 (Figure 2B) indicated that at least a part of the exosomes were targeted to the lysosome.

In addition other inhibitors have been tested. First, cells incubated with 5 μg/ml cytochalasin D, which is known to inhibit actin polymerization and consequently inhibit phagocytosis [26] as well as other endocytic pathways [27] showed an uptake reduction of 32 ± 7% (n = 4) (Figure 2C). EIPA at 50 μM, which is known to block macropinocytosis [28], caused an uptake reduction of 36 ± 13% (n = 5) (Figure 2C), thus suggesting that exosomes were internalized via macropinocytosis. Methyl-beta-cyclodextrin, that is used to deplete cholesterol from cellular membranes [29], decreased Exos-CFSE uptake (44 ± 8%, n = 5) (Figure 2C). However, there was no colocalization with caveolin-1 (Figure 2B), which is a marker of caveolae that are enriched in cholesterol rich domains. These results indicated that exosome uptake could occur via a cholesterol associated pathway independent of caveolae or that methyl-beta-cyclodextrin affected exosomes membrane integrity, thus decreasing uptake efficiency.

Exos-CFSE did not colocalize with the Golgi marker GM130, the *trans*-Golgi network marker TGN46 or the endoplasmic reticulum marker calnexin (data not shown).

### Proteins are required for exosomes uptake

**Figure 3:**
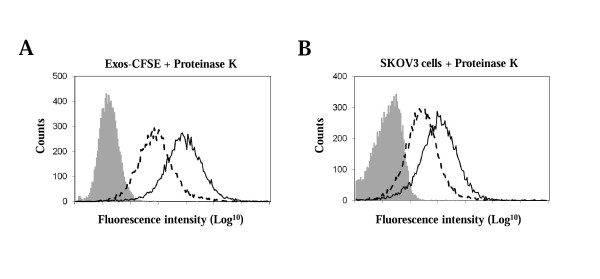
**Effect of proteinase K treatment in SKOV3 exosomes uptake**. (A) Exos-CFSE (20 μg protein; green) or (B) SKOV3 cells were treated with 100 μg/ml proteinase K for 30 min. Uptake was determined after 4 h of incubation by flow cytometry analysis and compared with uptake of Exos-CFSE with no treatment (solid lines). Unlabelled SKOV3 cells (grey) were used as control for cell autofluorescence. Dashed lines represent Exos-CFSE uptake after proteinase K digestion. The results shown are representative of three independent experiments performed in duplicate.

The recognition between exosomes and the target cell has been reported to involve proteins present at the cell surface of both exosomes and target cells [11,14]. Here, Exos-CFSE or SKOV3 cells were digested with proteinase K, a broad specificity protease, and uptake efficiency was analyzed by flow cytometry analysis. Uptake levels of digested Exos-CFSE were found to be lower (45 ± 12%, n = 6) than Exos-CFSE without treatment (Figure 3A). As control, fluorescence of Exos-CFSE was not affected by proteinase K treatment (data not shown). Furthermore, a decrease of 32 ± 8% (n = 6) in uptake was observed in SKOV3 cells treated with proteinase K (Figure 3B). Therefore, proteins present in exosomes and also in SKOV3 cells are required, at least in part, for internalization by target cells.

### Enrichment of specific glycoproteins in exosomes and relevance in uptake

Glycan-lectin interactions have been suggested to play a role in the uptake of exosomes by target cells [19,20], therefore, we have investigated if glycans would play a role in exosome uptake by SKOV3 cells.

**Figure 4:**
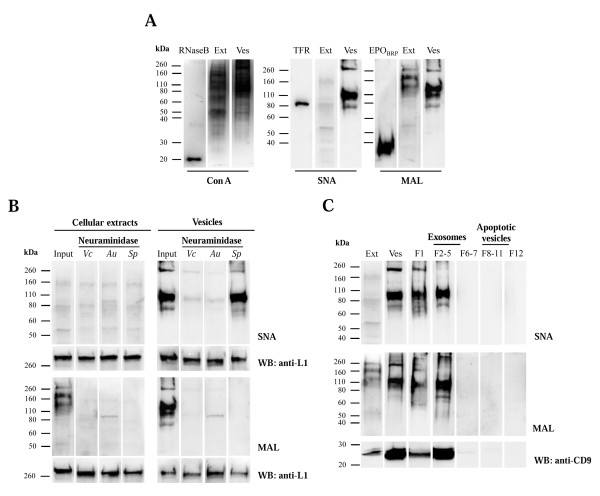
**Western blot and lectin detection of glycoproteins from SKOV3 cellular extracts (Ext) and secreted vesicles (Ves)**. (A) Con A, SNA and MAL lectin detection in SKOV3 cellular extract and vesicles. Three μg total protein were applied per lane. As positive controls, 200 ng ribonuclease B (RNase B) [41], human plasma transferrin (TFR) [42] and erythropoietin (EPO_BRP_) [43] were used. (B) SNA and MAL lectin analysis of desialylated SKOV3 cellular extracts and vesicles. Total proteins (3 μg) were digested with neuraminidases from *V. cholerae (Vb)*, *A. urefaciens (Au) *and *S. pneumonia (Sp)*. Input consisted of cellular extracts and exosomes without treatment. As loading control L1 was detected. (C) Vesicles from 1.5 × 10^7 ^SKOV3 cells were fractionated in a sucrose gradient. Cellular extracts (Ext), secreted vesicles from 100,000 × g pellet (Ves), pooled fractions 2-5 (F2-5) (3 μg total protein), 20% of F1, F6-7, F8-11 and F12 were analysed. As positive control for exosomes, CD9 was detected. Detection was performed using the chemiluminescent method.

First, glycoproteins of cellular extracts and secreted vesicles from SKOV3 cells were detected with the lectins concanavalin A (Con A; binds α-mannosyl containing-branched glycans predominantly of the high-mannose followed by hybrid- and biantennary complex type structures to a lower extent) [30], *Sambucus nigra *lectin (SNA; recognizes NeuAcα2,6Gal/GalNAc) and *Maackia amurensis *lectin (MAL; binds NeuAcα2,3Galβ1,4GlcNAc/Glc) [31]. The profile from exosomes was distinct from that of cellular extracts with the three lectins (Figure 4A). Detection with SNA and MAL was almost totally abolished after digestion with *V. Cholerae *and *A. urefaciens *neuraminidases (cleave terminal *α*2,3- and *α*2,6-linked NeuAc), whereas only MAL binding was abolished after *S. pneumoniae *(cleaves only *α*2,3-linked NeuAc) digestion (Figure 4B), thus confirming that SNA and MAL binding to secreted vesicles glycoproteins was specific. The sialic acid was not present in sialylated Lewis epitopes, since there was no detection with anti-sialyl-Lewis^a ^or anti- sialyl-Lewis^x ^antibodies (data not shown).

SKOV3 secreted vesicles are constituted by exosomes and apoptotic vesicles, which can be fractionated by using a sucrose gradient, as previously described [21,22]. CD9 is a tetraspanin protein that has been used as exosome marker [32,33]. After sucrose gradient separation of the secreted vesicles all the SNA and MAL binding was found in the exosome-containing fractions (fractions 2-5) and not in the apoptotic blebs (fractions 8-11) (Figure 4C).

**Figure 5:**
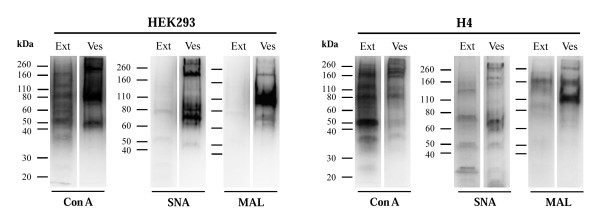
**Con A, SNA and MAL lectin detection of glycoproteins from HEK293 and H4 cellular extract (Ext) and secreted vesicles (Ves)**. For the analysis, 3 μg of total protein were applied per lane. Detection was performed using the chemiluminescent method.

Specific patterns of protein glycosylation were also found for exosomes from two other human cell lines, embryonic kidney HEK293 and neuroglioma H4 cells (Figure 5).

**Figure 6:**
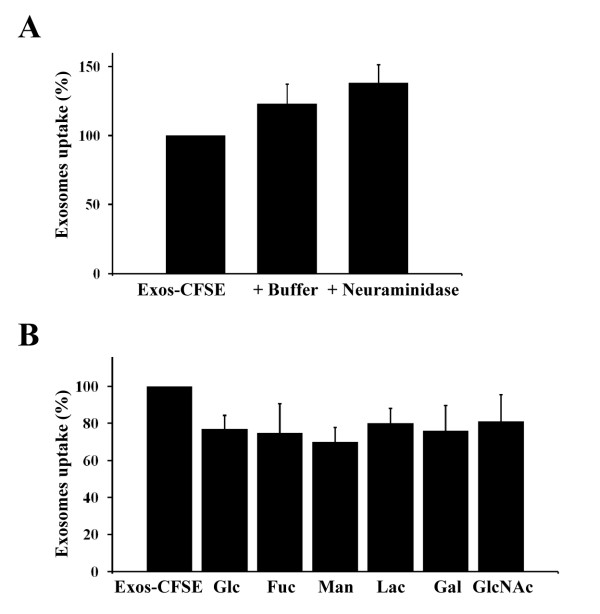
**Effects of neuraminidase and sugars on SKOV3 exosomes uptake**. (A) Exos-CFSE (20 μg protein) were incubated with 15 mU *V. cholerae *neuraminidase or corresponding buffer for 2 h before Exos-CFSE uptake. (B) Effect of 150 mM D-glucose (Glc), α-L-fucose (Fuc), α-D-mannose (Man), β-lactose (Lac), D-galactose (Gal) and D-*N*-acetylglucosamine (GlcNAc) (30 min pre-incubation) on Exos-CFSE uptake (4 h) monitored by flow cytometry analysis. Uptake efficiency was calculated relatively to the uptake without treatment, considered as 100%. Results are displayed in relative percentages ± S.D. The results shown are representative of three independent experiments performed in duplicate.

To investigate a possible role for glycosylation in exosome uptake Exos-CFSE were desialylated with *V. cholerae *and *A. urefaciaens *sialidases and exosomes uptake was monitored by immunofluorescence microscopy and flow cytometry analysis of cell fluorescence intensity. Uptake efficiency of exosomes after neuraminidase treatment was slightly increased (16 ± 14%, n = 6) when compared with exosomes incubated with neuraminidase buffer (Figure 6A), however, the observed increase was not statistically significant using the Student *t *test (P = 0.0764). In addition, increases in the uptake of exosomes incubated with neuraminidase (38 ± 13%, n = 6) or neuraminidase buffer (23 ± 14%, n = 6) were detected when compared with Exos-CFSE without treatment (Figure 6A).

The uptake assay was also performed in the presence of 150 mM of the monosaccharides D-glucose (control), D-galactose, α-L-fucose, α-D-mannose and D-*N*-acetylglucosamine, and the disaccharide β-lactose. Decreases of 23 ± 7%, 24 ± 14%, 25 ± 16%, 27 ± 8%, 19 ± 15% and 20 ± 8% (n = 6), respectively, in the uptake of Exos-CFSE in comparison with control without sugar were observed. The incubation with α-D-mannose led to a higher decrease in uptake relatively to the control sugar D-glucose, however the difference was not statistically significant (Figure 6B).

## Discussion

Exosomes are small membrane vesicles that are secreted by several cell types, including tumors and they have been found in biological fluids. They contain several membrane and cytoplasmic proteins and, in cancer, they play a role in cell migration and metastases. They may also transfer proteins associated with deregulated signalling pathways in cancer [34,35], therefore, contributing to the propagation of transformed phenotype. Furthermore, the exosomes may transfer mRNA and miRNA [36].

In the present work, we have found that the SKOV3 ovarian carcinoma cell line internalizes exosomes derived from the same cells in an energy-dependent process, via various endocytic pathways: colocalization studies with organelle markers and incubation with inhibitors have shown that the endocytosis pathway dependent on clathrin, macropinocytosis and a cholesterol associated pathway independent of caveolae were associated with the uptake. Evidence from the literature showed that in dendritic cells exosome uptake was also inhibited at 4°C and with cytochalasin D, and was further trafficked to the late endosomes/lysosomes [14]. In phagocytic cells, more specifically macrophages, internalization was also dependent on the actin cytoskeleton but not on pathways involving caveolae, macropinocytosis or clathrin-coated vesicles [15]. In the PC12 cell line exosomes uptake was also found to occur via the endocytic pathway [27]. After internalization, exosomes could fuse with the endosomal membrane and deliver their content to the cytoplasm of the target cell. For SKOV3 cells such process could take place over a long period of time since internalized exosomes were detected in the cells for at least 20 h (data not shown).

The initial recognition events that precede uptake may involve several molecules, proteins [16] and lipids [12] have already been indicated as involved in this process. In this work, the impairment observed for vesicles or cells treated with proteinase K indicated that proteins from the extracellular surface from both target cells and exosomes are required for internalization. Further studies to identify which proteins play an important role in the recognition are required.

Here we also found that exosomes from SKOV3 cells are particularly enriched in specific glycoproteins with high-mannose or NeuAcα2,3/6-containing structures. In this context, other authors, using lectin arrays, have also observed the enrichment in T-cells secreted microvesicles of high-mannose and sialic acid-containing structures in comparison with cell membranes [18]. Furthermore, an enrichment of more extensively glycosylated forms of PrPc has been found in the exosomes in comparison with cell lysates [37].

Removal of NeuAc from the exosomes with neuraminidase led to an increase in the uptake, but non-significant. The removal of NeuAc decreased the negative charge at the exosome surface exposing galactose or N-acetylgalactosamine residues. This could lead to physico-chemical alterations of the membrane or create new ligands for carbohydrate binding proteins at the surface of the cells that would mediate the binding.

The monosaccharides D-galactose, α-L-fucose, α-D-mannose, D-N-acetylglucosamine and the disaccharide β-lactose reduced exosomes uptake to a comparable extent as the control D-glucose probably due to increased osmotic pressure that is known to reduce endocytosis [38]. Therefore, these sugars do not play a major role in exosome interaction and uptake in SKOV3 cells. This result is different from that found in dendritic cells where exosome uptake was specifically inhibited by mannose and N-acetylglucosamine, the interaction being at least in part mediated by a C-type lectin [19], or in macrophages where lactose diminished exosome uptake probably through its action on galectin-5 that mediated the process [20].

Here, certain glycoproteins were specifically detected in exosomes and may constitute markers. It will be interesting to investigate if those glycoproteins are selectively sorted to the exosomes or if only certain glycoforms from the same protein are sorted into the exosomes. It can be admitted that glycans by themselves may be important for glycoprotein sorting into exosomes. Since protein oligomerization is known to promote sorting into exosomes independently of the ESCRT machinery and ubiquitination [16], the glycans could mediate oligomerization via interaction with lectins as previously described for the transferrin receptor in reticulocytes [39]. Moreover, glycans may interact with galectins at the cell surface, which may lead to their enrichment in membrane lipid domains as previously described [40]. Considering that lipid microdomains play a role on exosomes biogenesis [16], glycans could in that case also have a role on sorting to exosomes. Further studies are required to clarify this matter.

Exosomes contain cell surface cancer antigens, which confers them the potential for therapeutic approaches in cancer vaccination. Our observation that they are particularly enriched in glycan epitopes urges the need to further characterize the corresponding structures since one of the major changes that occur in cancer is in cell surface glycosylation, which has been largely used as biomarker. The knowledge of exosomes glycosylation with the possibility for its modulation will open new perspectives in cancer vaccination.

## Conclusions

Exosomes secreted by the ovarian cancer SKOV3 cell line are internalized by the same cells, and internalization is energy-dependent and it occurs via various endocytic pathways. Moreover, the interaction requires proteins from the cells and the exosomes. On the other hand, the exosomes were found to be enriched in specific mannose- and sialic acid-containing glycoproteins that could constitute exosome markers. Furthermore, sialic acid removal caused a small though non-significant increase in uptake.

## Abbreviations

Con A: Concanavalin A; SNA: *Sambucus nigra *lectin; MAL: *Maackia amurensis *lectin; CFSE: carboxyfluoresceine diacetate succinimidyl-ester; Exos: exosomes; EIPA: 5-ethyl-N-isopropyl amiloride; NeuAc: N-acetylneuraminic acid.

## Competing interests

The authors declare that they have no competing interests.

## Authors' contributions

CE contributed to study design, data interpretation, carried out the experiments and prepared the manuscript. SK contributed to experimental work and data interpretation. PA contributed to study design, data interpretation and manuscript revision. JC coordinated the study design, data interpretation and manuscript revision. All authors have read and approved the final manuscript.

## Pre-publication history

The pre-publication history for this paper can be accessed here:

http://www.biomedcentral.com/1471-2407/11/108/prepub

## References

[B1] Denzer K Kleijmeer MJ Heijnen HF Stoorvogel W Geuze HJ Exosome: from internal vesicle of the multivesicular body to intercellular signaling device J Cell Sci 2000 113 19 3365 3374 10984428 10.1242/jcs.113.19.3365

[B2] Keller S Sanderson MP Stoeck A Altevogt P Exosomes: From biogenesis and secretion to biological function Immunol Lett 2006 107 2 102 108 10.1016/j.imlet.2006.09.005 17067686

[B3] Simpson RJ Lim JWE Moritz RL Mathivanan S Exosomes: proteomic insights and diagnostic potential Expert Rev Proteomic 2009 6 3 267 283 10.1586/epr.09.17 19489699

[B4] van Niel G Porto-Carreiro I Simoes S Raposo G Exosomes: A Common Pathway for a Specialized Function J Biochem 2006 140 1 13 21 10.1093/jb/mvj128 16877764

[B5] Katzmann DJ Babst M Emr SD Ubiquitin-Dependent Sorting into the Multivesicular Body Pathway Requires the Function of a Conserved Endosomal Protein Sorting Complex, ESCRT-I Cell 2001 106 2 145 155 10.1016/S0092-8674(01)00434-2 11511343

[B6] Lakkaraju A Rodriguez-Boulan E Itinerant exosomes: emerging roles in cell and tissue polarity Trends Cell Biol 2008 18 5 199 209 10.1016/j.tcb.2008.03.002 18396047 PMC3754907

[B7] de Gassart A Geminard C Fevrier B Raposo G Vidal M Lipid raftassociated protein sorting in exosomes Blood 2003 102 13 4336 4344 10.1182/blood-2003-03-0871 12881314

[B8] Schorey JS Bhatnagar S Exosome Function: From Tumor Immunology to Pathogen Biology Traffic 2008 9 6 871 881 10.1111/j.1600-0854.2008.00734.x 18331451 PMC3636814

[B9] Trajkovic K Hsu C Chiantia S Rajendran L Wenzel D Wieland F Schwille P Brugger B Simons M Ceramide Triggers Budding of Exosome Vesicles into Multivesicular Endosomes Science 2008 319 5867 1244 1247 10.1126/science.1153124 18309083

[B10] Ostrowski M Carmo NB Krumeich S Fanget I Raposo G Savina A Moita CF Schauer K Hume AN Freitas RP Rab27a and Rab27b control different steps of the exosome secretion pathway Nat Cell Biol 2010 12 1 19 30 10.1038/ncb2000 19966785

[B11] Thery C Ostrowski M Segura E Membrane vesicles as conveyors of immune responses Nat Rev Immunol 2009 9 8 581 593 10.1038/nri2567 19498381

[B12] Keller S König A-K Marme F Runz S Wolterink S Koensgen D Mustea A Sehouli J Altevogt P Systemic presence and tumor-growth promoting effect of ovarian carcinoma released exosomes Cancer Lett 2009 278 1 73 81 10.1016/j.canlet.2008.12.028 19188015

[B13] Cocucci E Racchetti G Meldolesi J Shedding microvesicles: artefacts no more Trends in Cell Biol 2009 19 2 43 51 10.1016/j.tcb.2008.11.003 19144520

[B14] Morelli AE Larregina AT Shufesky WJ Sullivan MLG Stolz DB Papworth GD Zahorchak AF Logar AJ Wang Z Watkins SC Endocytosis, intracellular sorting, and processing of exosomes by dendritic cells Blood 2004 104 10 3257 3266 10.1182/blood-2004-03-0824 15284116

[B15] Feng D Zhao W-L Ye Y-Y Bai X-C Liu R-Q Chang L-F Zhou Q Sui S-F Cellular Internalization of Exosomes Occurs Through Phagocytosis Traffic 2010 11 5 675 687 10.1111/j.1600-0854.2010.01041.x 20136776

[B16] Simons M Raposo G Exosomes--vesicular carriers for intercellular communication Curr Opin Cell Biol 2009 21 4 575 581 10.1016/j.ceb.2009.03.007 19442504

[B17] Stoorvogel W Kleijmeer MJ Geuze HJ Raposo G The Biogenesis and Functions of Exosomes Traffic 2002 3 5 321 330 10.1034/j.1600-0854.2002.30502.x 11967126

[B18] Krishnamoorthy L Bess JW Preston AB Nagashima K Mahal LK HIV-1 and microvesicles from T cells share a common glycome, arguing for a common origin Nat Chem Biol 2009 5 4 244 250 10.1038/nchembio.151 19234452 PMC2713040

[B19] Hao S Bai O Li F Yuan J Laferte S Xiang J Mature dendritic cells pulsed with exosomes stimulate efficient cytotoxic T-lymphocyte responses and antitumour immunity Immunology 2007 120 1 90 102 10.1111/j.1365-2567.2006.02483.x 17073943 PMC2265880

[B20] Barres C Blanc L Bette-Bobillo P Andre S Mamoun R Gabius H-J Vidal M Galectin-5 is bound onto the surface of rat reticulocyte exosomes and modulates vesicle uptake by macrophages Blood 2010 115 3 696 705 10.1182/blood-2009-07-231449 19903899

[B21] Stoeck A Keller S Riedle S Sanderson MP Runz S Le Naour F Gutwein P Ludwig A Rubinstein E Altevogt P A role for exosomes in the constitutive and stimulus-induced ectodomain cleavage of L1 and CD44 Biochem J 2006 393 Pt 3 609 618 16229685 10.1042/BJ20051013 PMC1360713

[B22] Escrevente C Morais VA Keller S Soares CM Altevogt P Costa J Functional role of N-glycosylation from ADAM10 in processing, localization and activity of the enzyme BBA - Gen Subjects 2008 1780 6 905 913 10.1016/j.bbagen.2008.03.004 18381078

[B23] Parish CR Fluorescent dyes for lymphocyte migration and proliferation studies Immunol Cell Biol 1999 77 6 499 508 10.1046/j.1440-1711.1999.00877.x 10571670

[B24] Doherty GJ McMahon HT Mechanisms of Endocytosis Annu Rev Biochem 2009 78 1 857 902 10.1146/annurev.biochem.78.081307.110540 19317650

[B25] Wang LH Rothberg KG Anderson RG Mis-assembly of clathrin lattices on endosomes reveals a regulatory switch for coated pit formation J Cell Biol 1993 123 5 1107 1117 10.1083/jcb.123.5.1107 8245121 PMC2119875

[B26] DeFife KM Jenney CR Colton E Anderson JM Disruption of filamentous actin inhibits human macrophage fusion FASEB J 1999 13 8 823 832 10224226 10.1096/fasebj.13.8.823

[B27] Tian T Wang Y Wang H Zhu Z Xiao Z Visualizing of the cellular uptake and intracellular trafficking of exosomes by live-cell microscopy J Cell Biochem 2010 20533300 10.1002/jcb.22733

[B28] West MA Bretscher MS Watts C Distinct endocytotic pathways in epidermal growth factor-stimulated human carcinoma A431 cells J Cell Biol 1989 109 6 2731 2739 10.1083/jcb.109.6.2731 2556406 PMC2115909

[B29] Rodal SK Skretting G Garred O Vilhardt F van Deurs B Sandvig K Extraction of Cholesterol with Methyl-beta-Cyclodextrin Perturbs Formation of Clathrin-coated Endocytic Vesicles Mol Biol Cell 1999 10 4 961 974 10198050 10.1091/mbc.10.4.961 PMC25220

[B30] Bhattacharyya L Brewer CF Interactions of concanavalin A with asparagine-linked glycopeptides Eur J Biochem 1989 178 3 721 726 10.1111/j.1432-1033.1989.tb14503.x 2912731

[B31] Knibbs RN Goldstein IJ Ratcliffe RM Shibuya N Characterization of the carbohydrate binding specificity of the leukoagglutinating lectin from Maackia amurensis. Comparison with other sialic acid-specific lectins J Biol Chem 1991 266 1 83 88 1985926

[B32] Fevrier B Raposo G Exosomes: endosomal-derived vesicles shipping extracellular messages Curr Opin Cell Biology 2004 16 4 415 421 10.1016/j.ceb.2004.06.003 15261674

[B33] Lamparski HG Metha-Damani A Yao J-Y Patel S Hsu D-H Ruegg C Le Pecq J-B Production and characterization of clinical grade exosomes derived from dendritic cells J Immunol Methods 2002 270 2 211 226 12379326 10.1016/s0022-1759(02)00330-7

[B34] Al-Nedawi K Meehan B Micallef J Lhotak V May L Guha A Rak J Intercellular transfer of the oncogenic receptor EGFRvIII by microvesicles derived from tumour cells Nat Cell Biol 2008 10 5 619 624 10.1038/ncb1725 18425114

[B35] Iero M Valenti R Huber V Filipazzi P Parmiani G Fais S Rivoltini L Tumour-released exosomes and their implications in cancer immunity Cell Death Differ 2007 15 1 80 88 10.1038/sj.cdd.4402237 17932500

[B36] Skog J Wurdinger T van Rijn S Meijer DH Gainche L Curry WT Carter BS Krichevsky AM Breakefield XO Glioblastoma microvesicles transport RNA and proteins that promote tumour growth and provide diagnostic biomarkers Nat Cell Biol 2008 10 12 1470 1476 10.1038/ncb1800 19011622 PMC3423894

[B37] Vella LJ Sharples RA Lawson VA Masters CL Cappai R Hill AF Packaging of prions into exosomes is associated with a novel pathway of PrP processing J Pathol 2007 211 5 582 590 10.1002/path.2145 17334982

[B38] Oka JA Christensen MD Weigel PH Hyperosmolarity inhibits galactosyl receptor-mediated but not fluid phase endocytosis in isolated rat hepatocytes J Biol Chem 1989 264 20 12016 12024 2545695

[B39] Vidal M Mangeat P Hoekstra D Aggregation reroutes molecules from a recycling to a vesicle-mediated secretion pathway during reticulocyte maturation J Cell Sci 1997 110 16 1867 1877 9296387 10.1242/jcs.110.16.1867

[B40] Garner OB Baum LG Galectin-glycan lattices regulate cell-surface glycoprotein organization and signalling Biochem Soc Trans 2008 36 Pt 6 1472 1477 10.1042/BST0361472 19021578 PMC2811491

[B41] Fu D Chen L O'Neill RA A detailed structural characterization of ribonuclease B oligosaccharides by 1 H NMR spectroscopy and mass spectrometry Carbohyd Res 1994 261 2 173 186 10.1016/0008-6215(94)84015-6 7954510

[B42] Spik G Bayard B Fournet B Strecker G Bouquelet S Montreuil J Studies on glycoconjugates. LXIV. Complete structure of two carbohydrate units of human serotransferrin FEBS Lett 1975 50 3 296 299 1116600 10.1016/0014-5793(75)80513-8

[B43] Sasaki H Ochi N Dell A Fukuda M Site-specific glycosylation of human recombinant erythropoietin: analysis of glycopeptides or peptides at each glycosylation site by fast atom bombardment mass spectrometry Biochemistry 1988 27 23 8618 8626 10.1021/bi00423a017 3219367

